# Scirpy: a Scanpy extension for analyzing single-cell T-cell receptor-sequencing data

**DOI:** 10.1093/bioinformatics/btaa611

**Published:** 2020-07-02

**Authors:** Gregor Sturm, Tamas Szabo, Georgios Fotakis, Marlene Haider, Dietmar Rieder, Zlatko Trajanoski, Francesca Finotello

**Affiliations:** Biocenter, Institute of Bioinformatics, Medical University of Innsbruck, Innsbruck 6020, Austria; Biocenter, Institute of Bioinformatics, Medical University of Innsbruck, Innsbruck 6020, Austria; Biocenter, Institute of Developmental Immunology, Medical University of Innsbruck, Innsbruck 6020, Austria; Biocenter, Institute of Bioinformatics, Medical University of Innsbruck, Innsbruck 6020, Austria; Biocenter, Institute of Bioinformatics, Medical University of Innsbruck, Innsbruck 6020, Austria; Biocenter, Institute of Bioinformatics, Medical University of Innsbruck, Innsbruck 6020, Austria; Biocenter, Institute of Bioinformatics, Medical University of Innsbruck, Innsbruck 6020, Austria; Biocenter, Institute of Bioinformatics, Medical University of Innsbruck, Innsbruck 6020, Austria

## Abstract

**Summary:**

Advances in single-cell technologies have enabled the investigation of T-cell phenotypes and repertoires at unprecedented resolution and scale. Bioinformatic methods for the efficient analysis of these large-scale datasets are instrumental for advancing our understanding of adaptive immune responses. However, while well-established solutions are accessible for the processing of single-cell transcriptomes, no streamlined pipelines are available for the comprehensive characterization of T-cell receptors. Here, we propose **s**ingle-**c**ell **i**mmune **r**epertoires in **Py**thon (*Scirpy)*, a scalable Python toolkit that provides simplified access to the analysis and visualization of immune repertoires from single cells and seamless integration with transcriptomic data.

**Availability and implementation:**

*Scirpy* source code and documentation are available at https://github.com/icbi-lab/scirpy.

**Supplementary information:**

Supplementary data are available at *Bioinformatics* online.

## 1 Introduction

B and T lymphocytes are equipped with a vast repertoire of immune cell receptors that can recognize a wealth of different antigens. High-throughput sequencing technologies have enabled the study of these immune repertoires at unprecedented resolution (Finotello *et al.*, 2019; Hackl *et al.*, 2016) and are advancing our understanding of adaptive immune responses in cancer (Valpione *et al.*, 2020), as well as in autoimmune (Hanson *et al.*, 2020) and infectious (Schober *et al.*, 2020) diseases.

Novel single-cell sequencing technologies now allow the joint profiling of transcriptomes and T-cell receptors (TCRs) in single cells. However, while the study of single-cell transcriptomes is facilitated by tools like Seurat (Butler *et al.*, 2018) and Scanpy (Wolf *et al.*, 2018), the bioinformatic analysis of paired *α* and *β* TCR chains is still in its infancy. Several methods to perform specific analytical tasks have been proposed (Supplementary Table S1), but the comprehensive characterization of TCR diversity from single cells is still hampered by the lack of ready-to-use computational pipelines.

Here, we present *Scirpy* (**s**ingle-**c**ell **i**mmune **r**epertoires in **Py**thon), a Python-based Scanpy extension that provides simplified access to various computational modules for the analysis and visualization of immune repertoires from single cells. Due to its tight integration with Scanpy, *Scirpy* allows the combination with scRNA-seq transcriptomic data to comprehensively characterize the phenotype and TCR of single T cells.

## 2 The *Scirpy* package

**Fig. 1.**
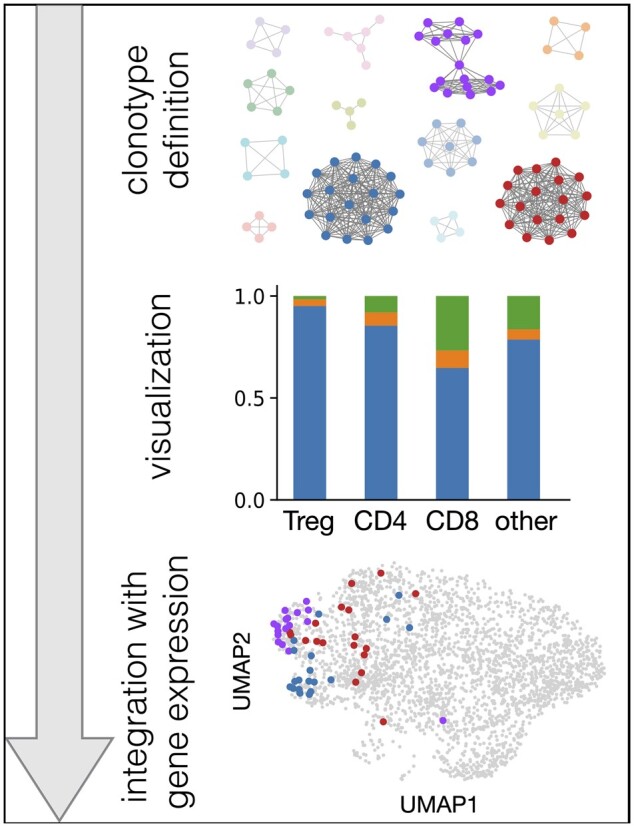
Schematization of the *Scirpy* workflow. Definition of clonotype networks (top panel), clonotype analysis and visualization (e.g. clonal expansion of T-cell subpopulations, middle panel), and integration with gene expression data (e.g. UMAP plot, bottom panel)

*Scirpy* integrates different bioinformatic methods for importing, analyzing and visualizing single-cell TCR-sequencing data from human and mouse (Fig. 1). TCR data can be loaded from CellRanger (10× Genomics) csv or json files, TraCeR (Stubbington *et al.*, 2016) outputs generated from Smart-seq2 data or any delimited text file, including AIRR-compliant tsv files (Vander Heiden *et al.*, 2018). The AnnData data structure provided by Scanpy is used to store TCR information together with matched transcriptomic profiles, when available.

*Scirpy* uses a flexible TCR model supporting up to two *α* and *β* chains per cell, allowing the identification of *dual-TCR T cells* (Schuldt and Binstadt, 2019) (Supplementary Note S1). It also flags cells with more than two chains, which potentially represent doublets (Supplementary Fig. S1) and may be discarded from downstream analyses. *Scirpy* defines *clonotypes* based on the nucleotide sequence of the TCR complementarity-determining region 3 (CDR3), but can further identify *clonotype clusters* based on CDR3 amino acid sequence identity or similarity. The latter approach, inspired by TCRdist (Dash *et al.*, 2017), leverages the Parasail library (Daily, 2016) to compute pairwise sequence alignments and identify clusters of T cells that likely recognize the same antigens. For building clonotype networks, *Scirpy* makes use of the sparse-matrix implementation from the scipy package (Virtanen *et al.*, 2020), ensuring scalability to hundreds of thousands of cells (Supplementary Fig. S2).

*Scirpy* offers a wide range of tools and visualization options that we demonstrate in Section 3. It allows inspecting TCR chain configurations (Supplementary Fig. S1), and exploring the abundance, diversity, expansion and overlap of clonotype repertoires across samples, patients or cell clusters derived from transcriptomics data (Supplementary Figs S3 and S4). Relationships between cells and clonotypes can be investigated with a graph-based approach (Supplementary Fig. S5), in addition to spectratype plots (which represent the distribution of CDR3 sequence lengths), and V(D)J-usage plots (Supplementary Fig. S6). Finally, TCR information can be integrated with transcriptomic data, for instance by overlaying Uniform Manifold Approximation and Projection (UMAP) plots (Becht *et al.*, 2019; Supplementary Fig. S4). Detailed tutorials on data loading and analysis with *Scirpy* are available at: https://icbi-lab.github.io/scirpy/tutorials.html.

## 3 Case study: re-analysis of 140k single T cells

To demonstrate the applicability to a real-world scenario, we re-analyzed a recent single-cell dataset of ∼140k T cells (Wu *et al.*, 2020). Single T cells were isolated from tumor, normal adjacent tissue, and peripheral blood of 14 patients with four different cancer types, and subjected to single-cell RNA and TCR sequencing with the 10× technology. Consistently with the original results, we found that the majority of clonotypes were singletons and only 9–18% of patients’ clonotypes were clonally expanded (Supplementary Fig. S3). Our results further confirm that ${\text{CD}8}^{+}$ effector, effector memory and tissue resident T cells comprised a large fraction of clonotypes that were expanded in both the tumor and normal tissue, while ${\text{CD}4}^{+}$ T cells consisted mostly of singletons (Supplementary Fig. S4). Moreover, leveraging *Scirpy’*s capability to group cells based on CDR3 sequence-similarity, we identified clonotype clusters indicating convergent TCR evolution (Supplementary Fig. S5). The analysis ran in 13 min on a single core of an Intel E5-2699A v4, 2.4 GHz CPU when defining clonotypes based on sequence identity, and in 42 min on 32 cores when using pairwise sequence alignment. A jupyter notebook to reproduce this case study is available at: https://icbi-lab.github.io/scirpy-paper/wu2020.html.

## 4 Conclusions

*Scirpy* is a versatile tool to analyze single-cell TCR-sequencing data that enables seamless integration with the Scanpy toolkit, the *de facto* standard for analyzing single-cell data in Python. *Scirpy* is highly scalable to big scRNA-seq data and, thus, allows the joint characterization of phenotypes and immune cell receptors in hundreds of thousands of T cells. An extension of *Scirpy* to characterize *γδ*-TCR and B-cell receptor repertoires is planned for the next release.

## Funding

This work was supported by the Austrian Science Fund (FWF) [project number T 974-B30 to F.F. and I3978 to Z.T.] and by the European Research Council (ERC) [advanced grant agreement number 786295 to Z.T.]. Z.T. is a member of the German Research Foundation (DFG) [project number TRR 241(INF)].

*Conflict of Interest*: none declared.

## Supplementary Material

btaa611_supplementary_data
