# Supplementary material for: Scirpy: a Scanpy extension for analyzing single-cell T-cell receptor-sequencing data
Source: Bioinformatics. 2020 Jul 2;36(18):4817–8. doi: 10.1093/bioinformatics/btaa611 (PMC7751015; doi:10.1093/bioinformatics/btaa611)
Supplement: btaa611_supplementary_data [file btaa611_supplementary_data.pdf]

Supplementary Information

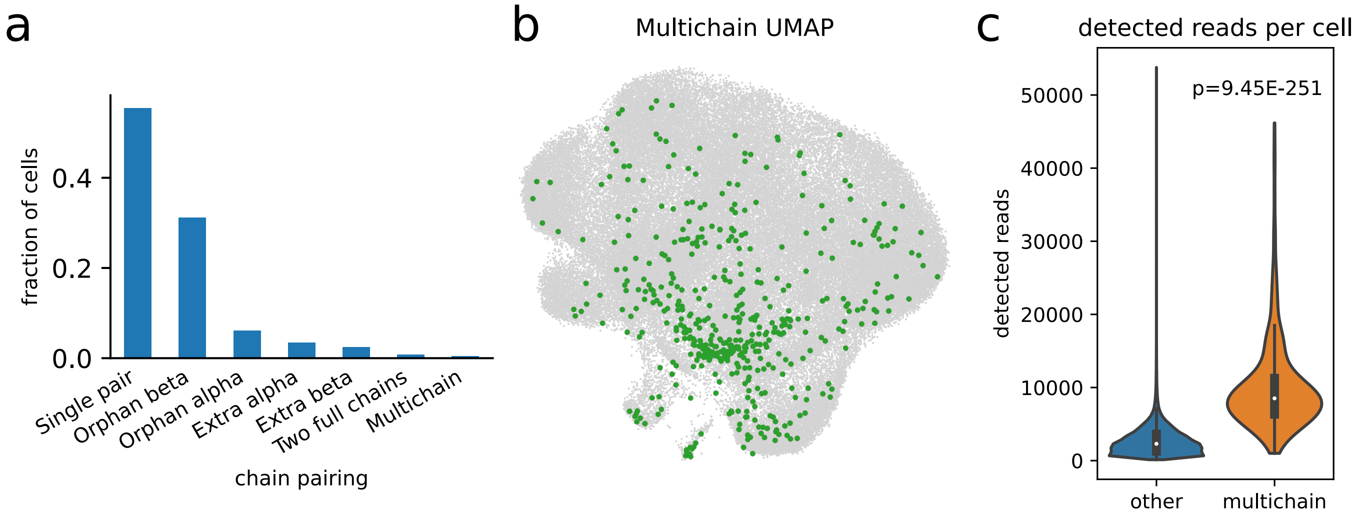

Supplementary Figure 1: Scirpy flags cells with more than two pairs of α and β TCR-chains as "multichain" cells. (a) The chart delineates the fraction of cells with a certain receptor configuration. "Orphan" alpha and beta designates cells that lost their β or α chains, respectively. "Extra" refers to cells having an extra chain in addition to a valid pair of α or β chains. **(b)** UMAP plot of 96,000 cells from Wu *et al.* [1] with at least one detected CDR3 sequence with multichain-cells (n=474) highlighted in green. **(c)** Comparison of detected reads per cell in multichain-cells and other cells. Multichain cells comprised significantly more reads per cell (median=8517) than other cells (median=2301, $p = 9.45 \times 10^{-251}$, Wilcoxon-Mann-Whitney-test), supporting the hypothesis that (most of) the multichain cells are technical artifacts arising from cell-multiplets [2].

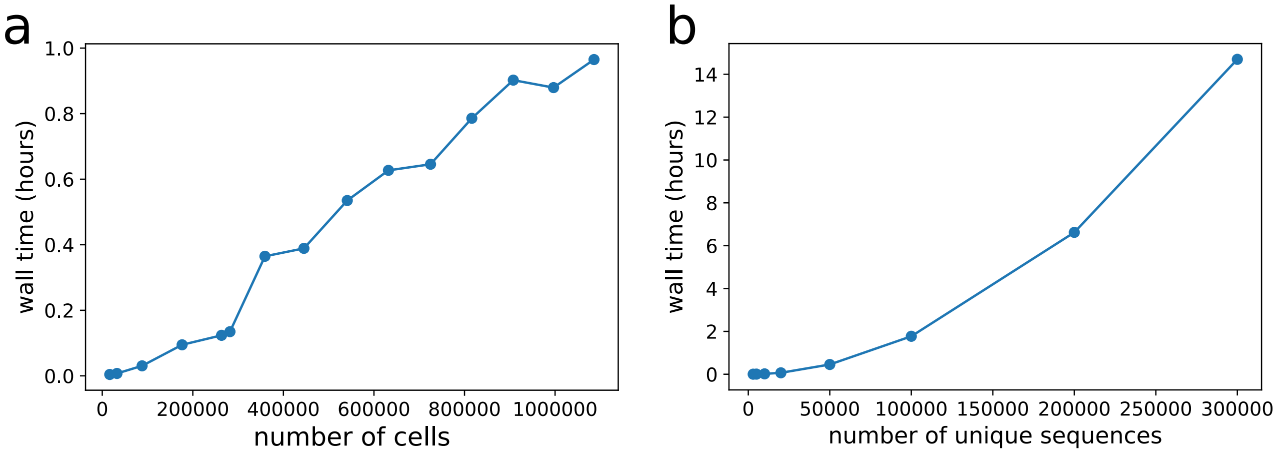

Supplementary Figure 2: Runtime analysis on simulated datasets. (a) Elapsed time for calculating the clonotype network based on nucleic acid sequence identity (`scirpy.tl.tcr_neighbors` function with `metric="identity"`)with increasing number of cells. The runtime is limited by the number of edges in the resulting cell $\times$ cell connectivity network. We simulated 500 000 α and β TCR sequences using the `immuneSIM` package [3], assuming a power law-distribution of clonal frequencies [3, 4]. To obtain clonal frequencies representative for single-cell TCR sequencing data, we fitted a power law distribution to the empirical distribution of clonotype frequencies in the Wu *et al.* [1] dataset using the `powerlaw` Python package [5]. From this distribution, we randomly sampled datasets with 5000 to 300 000 unique clonotypes, resulting in datasets with 16 553 to 1 085 999 cells. The analysis was performed on a single core of an Intel E5-2699A v4, 2.4 GHz CPU. **(b)** Elapsed time for calculating the sequence $\times$ sequence alignment-distance matrix on up to 300 000 simulated α TCR sequences. This distance-matrix is internally computed for α and β CDR3 amino-acid sequences independently when executing the `scirpy.tl.tcr_neighbors` function with `metric="alignment"`.The runtime is quadratic over the number of unique sequences and more computationally expensive than the sequence-identity metric due to the computation of pairwise sequence alignments. The analysis was performed on 16 cores of an Intel E5-2699A v4, 2.4 GHz CPU. The source-code to reproduce the runtime analysis is available from: https://github.com/icbi-lab/scirpy-paper/tree/master/runtime-analysis.

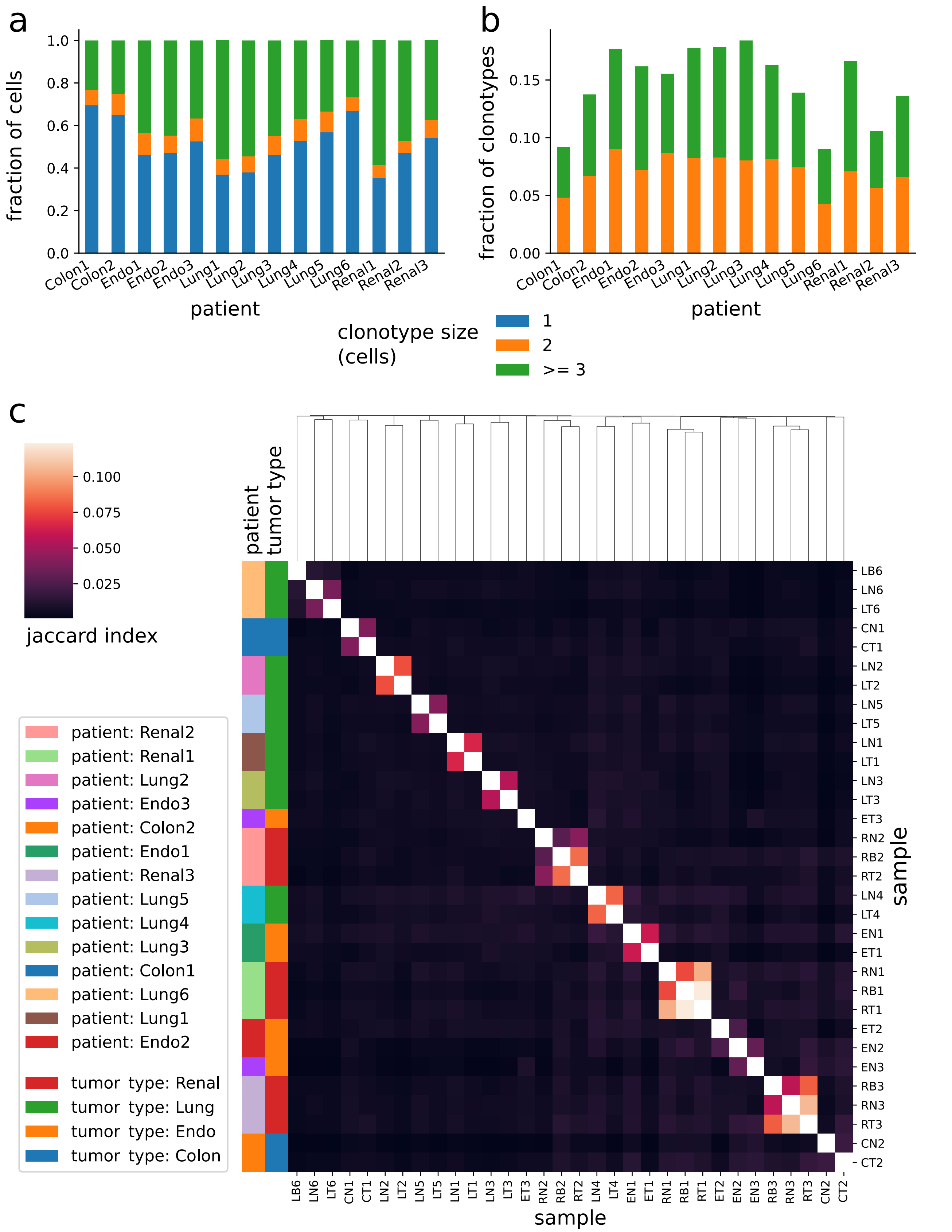

Supplementary Figure 3: T cell clonal expansion per patient. **(a)** Fraction of cells belonging to clonotypes with one cell (singletons, in blue), two cells (orange), or more than two cells (green). **(b)** Fraction of clonotypes comprising two, or more than two cells, respectively. Overall, between 9% and 18% of clonotypes were expanded, i.e. composed of more than one cell. **(c)** Overlap of clonotype clusters based on amino-acid sequence similarity between samples. Each tile of the heatmap represents the Jaccard-index between the respective pair of samples.

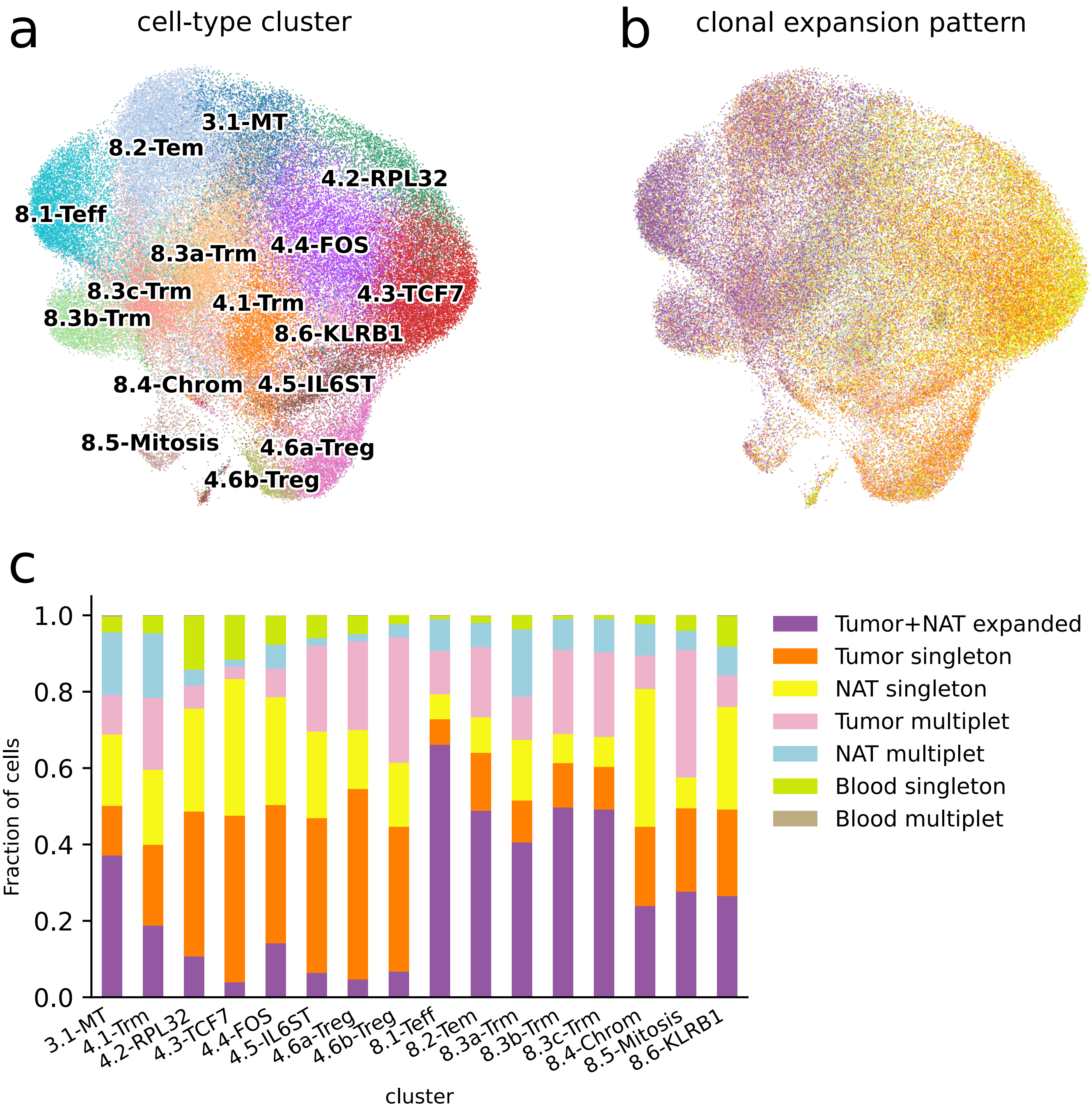

Supplementary Figure 4: Differential tissue expansion patterns by T-cell clusters. (a) UMAP plot of 96,000 cells from Wu *et al.* [1] with at least one detected CDR3 region, colored according to the cell-type clusters described in the original study. **(b, c)** Distribution of tissue expansion patterns by clustered T-cell subpopulations visualized as (b) UMAP plot and (c) bar chart. While $CD8^+$ effector, effector memory, and tissue-resident T-cells tended to be expanded in both tumor and normal adjacent tissue (NAT), $CD4^+$ T-cell clusters primarily comprised singleton clonotypes.

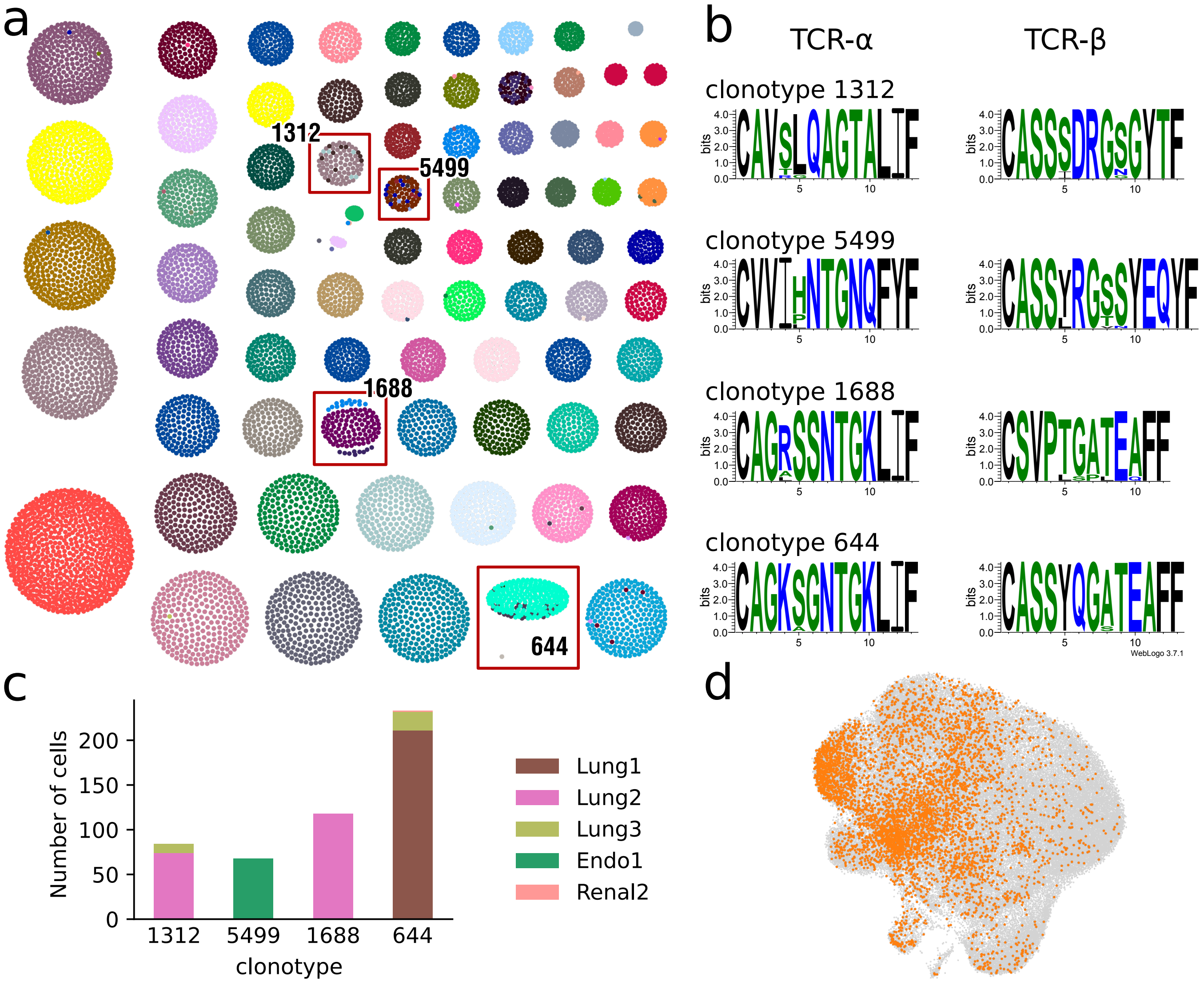

Supplementary Figure 5: Comparison of clonotypes with clonotype clusters based on sequence-similarity yields evidence of convergent evolution. **(a)** Clonotype clusters with more than 50 cells visualized as network plot. Each cluster represents a sub-network corresponding to a clonotype cluster defined based on amino-acid sequence similarity. Each dot represents an individual cell and is colored according to the clonotype assigned based on nucleotide-sequence identity. Four examples of clonotype clusters with heterogeneous nucleotide sequences are highlighted. Convergent clonotype clusters can be identified as subnetworks including different nucleotide sequences from the same patient. **(b)** Sequence logos of the primary TCR α and TCR β chains of the clonotypes highlighted in (a). **(c)** Patient composition of the four clonotypes highlighted in (a). Clonotype clusters number 5499 and 1688 comprised cells from a single patient only and were likely the result of convergent clonotype evolution. Clusters 1312 and 644 comprised cells from different patients and potentially target a common disease epitope (e.g. a viral epitope). **(d)** Cells from convergent clonotype clusters highlighted in the UMAP plot from Wu *et al.* [1].

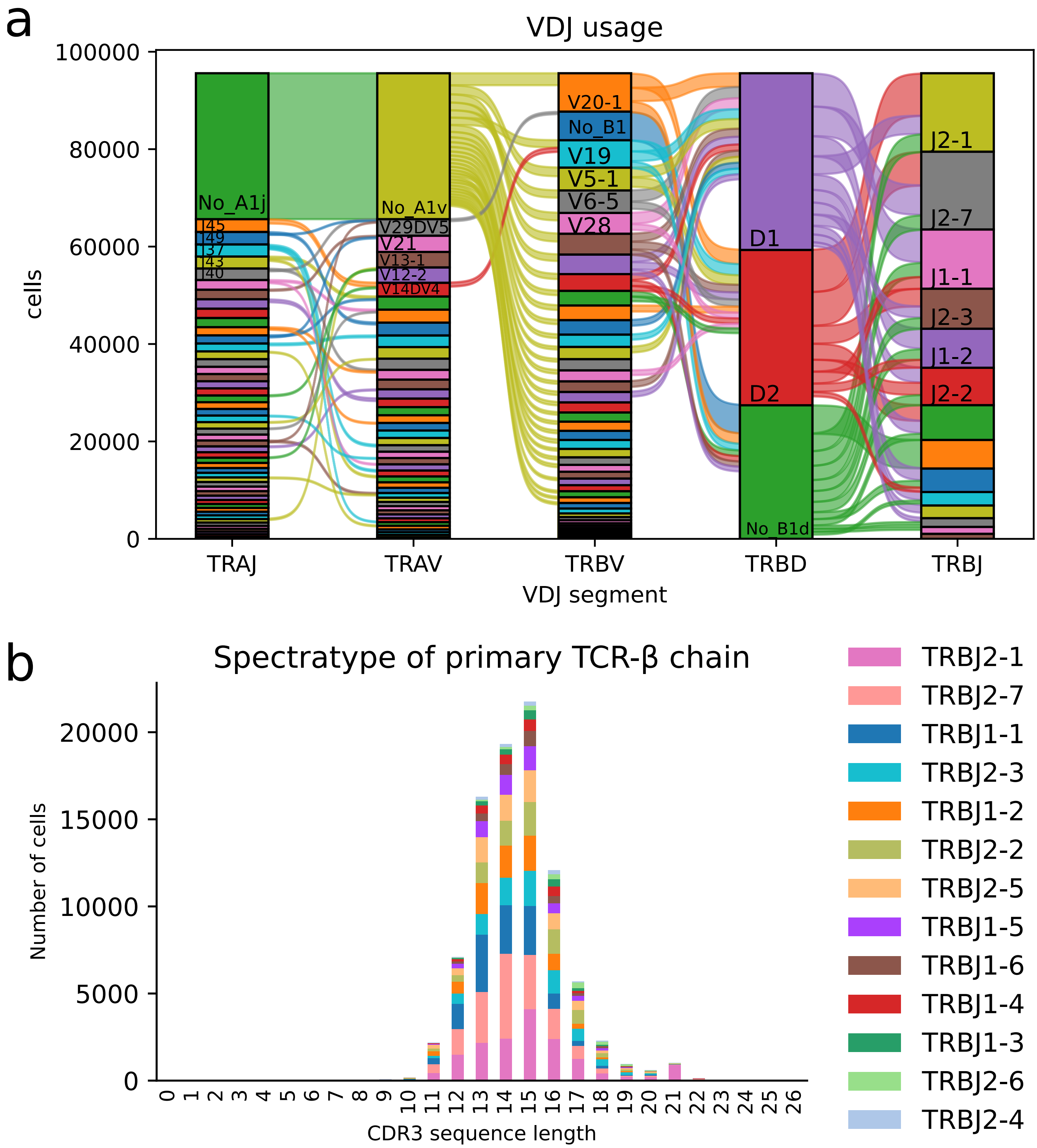

Supplementary Figure 6: V(D)J gene usage of 96,000 T cells from Wu *et al.* [1] **(a)** V(D)J gene usage of primary TCR α and β chains visualized as a Sankey plot. Each bar refers to a specific V(D)J segment, and each bar section to a specific V, D, or J gene. **(b)** Spectratype plot of the primary TCR β chain, colored by J gene usage. The bars show the distribution of TCR β CDR3 sequence length.

Tool name	Website	Language	Receptor type	Support for 10x	Support for Smart-seq2	Paired chains	Post-processing of clonotypes	Basic IR visualization	Advanced IR visualization	Clonotype clusters of single cells	Integrated with Gex	Ref.
Immcantation (pRESTO)	https://immcantation.readthedocs.io	Python (and R)	TCR BCR	✗	✗	✗	✓	✓	✓	✗	✗	[6]
ImmuneArch (tcR)	https://immunarch.com/	R	TCR BCR	✓	✗	✗	✓	✓	✓	✗	✗	[7]
iMonitor	https://github.com/zhangwei2015/IMonitor	Perl (and R)	TCR BCR	✗	✗	✗	✓	✓	✗	✗	✗	[8]
VDJtools	https://github.com/mikessh/vdjtools	Java (Python)	TCR BCR	✗	✗	✗	✓	✓	✓	✗	✗	[9]
TRUST4	https://github.com/liulab-dfci/TRUST4	Perl	TCR	(✓)[1]	(✓)[1]	✗	✗	✗	✗	✗	✗	[10]
scTCR Seq	https://github.com/ElementoLab/scTCRseq	Python	TCR	✗	✓	✓	✓	✓	✗	✗	✗	[11]
TraCeR	https://github.com/teichlab/tracer	Python	TCR	✗	✓	✓	✓	✓	✗	✓	(✓)[2]	[12]
VDJ Puzzle	https://github.com/simone-rizzetto/VDJPuzzle	Java	TCR	✗	✓	✗	✓	✓	✗	✗	(✓)[2]	[13]
TRAPes	https://github.com/YosefLab/TRAPeS	C++	TCR	✗	✓	✗	✗	✗	✗	✗	✗	[14]
Scirpy	https://github.com/icbi-lab/scirpy	Python	TCR	✓	✓	✓	✓	✓	✓	✓	✓	

Supplementary Table 1: Comparison of Scirpy with currently available tools supporting immune repertoire analysis at the single-cell level, or offering immune repertoire visualization. Basic immune repertoire (IR) visualization includes clonotype abundance, diversity, and V(D)J usage. Advanced IR visualization also includes repertoire overlap, clustering as well as specialized analysis of individual clonotypes and generation of publication-ready figures. “Gex” indicates gene expression data.

1. Supplementary Note

A *clonotype* designates a collection of T or B cells that descend from a common, antecedent cell, and therefore, bear the same adaptive immune receptors and recognize the same epitopes. In single-cell RNA-sequencing (scRNA-seq) data, T cells sharing identical complementarity-determining regions 3 (CDR3) nucleotide sequences of both α and β TCR chains are considered a clonotype.

Contrary to what would be expected based on the previously described mechanism of allelic exclusion [15], scRNA-seq datasets can feature a considerable number of cells with more than one TCR α and β pair. Since cells with more than one productive CDR3 sequence for each chain did not fit into common understanding of T cell biology, most TCR analysis tools ignore these cells [16, 17] or select the CDR3 sequence with the highest expression level [14]. While in some cases these double-TCR cells might represent artifacts (e.g. cell doublets), there is an increasing amount of evidence in support of a *bone fide* dual-TCR population [18, 19].

Scirpy allows investigating the composition and phenotypes of both single- and dual-TCR T cells by leveraging a T cell model similar to the one proposed in [12], where T cells are allowed to have a primary and a secondary pair of α and β chains. For each cell, the primary pair consists of the α and β chains with the highest read counts. Likewise, the secondary pair is the pair of α and β chains with the second highest read counts. Based on the assumption that each cell has only two copies of the underlying chromosome set, if more than two variants of a chain are recovered for the same cell, the excess TCR chains are ignored by Scirpy and the corresponding cells flagged as "multichain" (Supplementary Figure 1). Moreover, Scirpy flags as "orphan chain“ the cells that have lost their α or β chains, possibly due to sequencing inefficiencies resulting in chain dropouts. This filtering strategy leaves the choice of discarding or including multichain and orphan-chain cells in downstream analyses.

Scirpy implements a network-based clonotype analysis that enables clustering cells into clonotypes or clonotype clusters based on the following options:

(a) identical CDR3 nucleotide sequences;

(b) identical CDR3 amino acid sequences;

(c) similar CDR3 amino acid sequences based on pairwise sequence alignment.

The latter approach is inspired by studies showing that similar TCR sequences also share epitope targets [16, 20, 21]. While convergence of the nucleotide-based clonotype definition to the amino acid-based one hints at selection pressure, sequence alignment-based networks offer the opportunity to identify cells that might recognize the same epitopes.

Supplementary References

1. Wu, T. D. *et al.* Peripheral T cell expansion predicts tumour infiltration and clinical response. en. *Nature* (Feb. 2020).
2. Ilicic, T. *et al.* Classification of low quality cells from single-cell RNA-seq data. *Genome Biol.* **17,** 29 (Feb. 2016).
3. Weber, C. R. *et al.* immuneSIM: tunable multi-feature simulation of B- and T-cell receptor repertoires for immunoinformatics benchmarking. *Bioinformatics* (ed Schwartz, R.) `https://doi.org/10.1093/bioinformatics/btaa158` (Apr. 2020).
4. Greiff, V. *et al.* A bioinformatic framework for immune repertoire diversity profiling enables detection of immunological status. *Genome Medicine* **7.** `https://doi.org/10.1186/s13073-015-0169-8` (May 2015).
5. Alstott, J., Bullmore, E. & Plenz, D. powerlaw: A Python Package for Analysis of Heavy-Tailed Distributions. *PLoS ONE* **9** (ed Rapallo, F.) e85777. `https://doi.org/10.1371/journal.pone.0085777` (Jan. 2014).
6. Vander Heiden, J. A. *et al.* pRESTO: a toolkit for processing high-throughput sequencing raw reads of lymphocyte receptor repertoires. en. *Bioinformatics* **30,** 1930–1932 (July 2014).
7. Nazarov, V. I. *et al.* tcR: an R package for T cell receptor repertoire advanced data analysis. en. *BMC Bioinformatics* **16,** 175 (May 2015).
8. Zhang, W. *et al.* IMonitor: A Robust Pipeline for TCR and BCR Repertoire Analysis. en. *Genetics* **201,** 459–472 (Oct. 2015).
9. Shugay, M. *et al.* VDJtools: Unifying Post-analysis of T Cell Receptor Repertoires. en. *PLoS Comput. Biol.* **11,** e1004503 (Nov. 2015).
10. Li, B. *et al.* Landscape of tumor-infiltrating T cell repertoire of human cancers. en. *Nat. Genet.* **48,** 725–732 (July 2016).
11. Redmond, D., Poran, A. & Elemento, O. Single-cell TCRseq: paired recovery of entire T-cell alpha and beta chain transcripts in T-cell receptors from single-cell RNAseq. en. *Genome Med.* **8,** 80 (July 2016).
12. Stubbington, M. J. T. *et al.* T cell fate and clonality inference from single-cell transcriptomes. en. *Nat. Methods* **13,** 329–332 (Apr. 2016).
13. Rizzetto, S. *et al.* B-cell receptor reconstruction from single-cell RNA-seq with VDJPuzzle. en. *Bioinformatics* **34,** 2846–2847 (Aug. 2018).
14. Afik, S. *et al.* Targeted reconstruction of T cell receptor sequence from single cell RNA-seq links CDR3 length to T cell differentiation state. en. *Nucleic Acids Res.* **45,** e148 (Sept. 2017).
15. Brady, B. L., Steinel, N. C. & Bassing, C. H. Antigen receptor allelic exclusion: an update and reappraisal. en. *J. Immunol.* **185,** 3801–3808 (Oct. 2010).
16. Fischer, D. S., Wu, Y., Schubert, B. & Theis, F. J. Predicting antigen-specificity of single T-cells based on TCR CDR regions.
17. Zhang, L. *et al.* Lineage tracking reveals dynamic relationships of T cells in colorectal cancer. en. *Nature* **564,** 268–272 (Dec. 2018).
18. Schuldt, N. J. & Binstadt, B. A. Dual TCR T Cells: Identity Crisis or Multitaskers? en. *J. Immunol.* **202,** 637–644 (Feb. 2019).
19. Ji, Q., Perchellet, A. & Goverman, J. M. Viral infection triggers central nervous system autoimmunity via activation of CD8+ T cells expressing dual TCRs. en. *Nat. Immunol.* **11,** 628–634 (July 2010).
20. Glanville, J. *et al.* Identifying specificity groups in the T cell receptor repertoire. en. *Nature* **547,** 94–98 (July 2017).
21. Dash, P. *et al.* Quantifiable predictive features define epitope-specific T cell receptor repertoires. en. *Nature* **547,** 89–93 (July 2017).
